# Content and sentiment surveillance (CSI): A critical component for modeling modern epidemics

**DOI:** 10.3389/fpubh.2023.1111661

**Published:** 2023-03-16

**Authors:** Shi Chen, Shuhua Jessica Yin, Yuqi Guo, Yaorong Ge, Daniel Janies, Michael Dulin, Cheryl Brown, Patrick Robinson, Dongsong Zhang

**Affiliations:** ^1^Department of Public Health Sciences, College of Health and Human Services, University of North Carolina at Charlotte, Charlotte, NC, United States; ^2^School of Data Science, University of North Carolina at Charlotte, Charlotte, NC, United States; ^3^Academy for Population Health Innovation, University of North Carolina at Charlotte, Charlotte, NC, United States; ^4^Department of Software and Information Systems, College of Computing and Informatics, University of North Carolina at Charlotte, Charlotte, NC, United States; ^5^School of Social Work, College of Health and Human Services, University of North Carolina at Charlotte, Charlotte, NC, United States; ^6^Department of Bioinformatics and Genomics, College of Computing and Informatics, University of North Carolina at Charlotte, Charlotte, NC, United States; ^7^Department of Political Science and Public Administration, College of Liberal Arts and Sciences, University of North Carolina at Charlotte, Charlotte, NC, United States; ^8^Belk College of Business, University of North Carolina at Charlotte, Charlotte, NC, United States

**Keywords:** infoveillance, modeling, behavior, parameterization, mechanism, data-driven (DD)

## Abstract

Comprehensive surveillance systems are the key to provide accurate data for effective modeling. Traditional symptom-based case surveillance has been joined with recent genomic, serologic, and environment surveillance to provide more integrated disease surveillance systems. A major gap in comprehensive disease surveillance is to accurately monitor potential population behavioral changes in real-time. Population-wide behaviors such as compliance with various interventions and vaccination acceptance significantly influence and drive the overall epidemic dynamics in the society. Original infoveillance utilizes online query data (e.g., Google and Wikipedia search of a specific content topic such as an epidemic) and later focuses on large volumes of online discourse data about the from social media platforms and further augments epidemic modeling. It mainly uses number of posts to approximate public awareness of the disease, and further compares with observed epidemic dynamics for better projection. The current COVID-19 pandemic shows that there is an urgency to further harness the rich, detailed content and sentiment information, which can provide more accurate and granular information on public awareness and perceptions toward multiple aspects of the disease, especially various interventions. In this perspective paper, we describe a novel conceptual analytical framework of content and sentiment infoveillance (CSI) and integration with epidemic modeling. This CSI framework includes data retrieval and pre-processing; information extraction *via* natural language processing to identify and quantify detailed time, location, content, and sentiment information; and integrating infoveillance with common epidemic modeling techniques of both mechanistic and data-driven methods. CSI complements and significantly enhances current epidemic models for more informed decision by integrating behavioral aspects from detailed, instantaneous infoveillance from massive social media data.

## 1. Introduction

Mathematical models, such as the mechanistic susceptible exposed infectious recovered (SEIR) type modeling paradigm and alternative data-driven methods, have made investigations on epidemics across the globe (1). Epidemic modeling can systematically characterize epidemiological processes (e.g., transmission, immunization, hospitalization, recovery, etc.) and provide key metrics for epidemic projection, intervention, and resource optimization. In order to achieve these goals, a fundamental layer in epidemic modeling is to ensure comprehensive, accurate, and effective data collection through surveillance systems. The grand challenge of current epidemic modeling is to effectively identify, integrate, and analyze heterogeneous, cross-scale, and multimodal data from pathogen biology, human cognition and behavior, to social determinants of health (2).

Currently, many surveillance systems, such as the U.S. National Notifiable Diseases Surveillance System (NNDSS), have been developed from reported symptomatic cases. Additional surveillance systems, including genomic, serologic, and environmental surveillance systems in the CDC COVID-19 data dashboard, have been developed across national, state, and local levels, along with many other regions in the world (3, 4).

A key driver of epidemic dynamics is host cognition and behavior, such as adherence to interventions and vaccine acceptance. However, effective monitoring of behavior continuously on a large scale is challenging, as is quantifying its relevance to the observed health outcomes in an epidemic. Traditional participatory survey-based surveillance cannot provide comprehensive and continuous characterization of public perceptions toward the epidemic and various interventions, especially vaccination. Accurate characterization of public perceptions at different locations during different phases of an epidemic is critical to our efforts in designing and evaluating targeted interventions. To address this major issue, infoveillance, which observes, retrieves, and analyzes public online discourse especially on social media, has been developed since the 2000's (5–8). Infoveillance is implemented to monitor many diseases, including seasonal and pandemic influenza, Ebola, and COVID-19 epidemics (9–11). Traditional infoveillance approaches analyze online discourse dynamics of health issues by counting relevant posts and/or search queries. For instance, using COVID-19 specific terms, daily number or percentage of COVID-19-related posts and search queries can be counted. The discourse dynamics, expressed as the time series of the absolute number or relative percentage of the disease, is then compared with important health outcomes such as reported case, vaccination uptake, hospitalization, and death. Studies have shown that effective infoveillance can help predict early surges of an epidemic (9–11).

Nevertheless, we argue that traditional infoveillance—albeit offering advances in surveillance of various disease outbreaks, timing, and locations– lacks detailed extraction and characterization of dynamic public awareness, perceptions and sentiments toward interventions, which reflect behavioral changes and drive epidemic dynamics. Traditional infoveillance focuses on time series of posting counts or queries of the health issue, and ignores the large amount and rich information embedded in the actual contents of these discourses. With more recent advances in natural language processing (NLP), it is possible to further extract important information, such as contents and sentiments from social media posts (12–17, 20, 21). In this perspective paper, we introduce a conceptual framework of comprehensive content and sentiment infoveillance (CSI), including data mining and knowledge discovery of content and sentiment from social media discussions on epidemics (especially toward important interventions such as vaccination) with spatio-temporal variations, and integration with existing mechanistic and data-driven epidemic modeling techniques.

## 2. Content and sentiment infoveillance framework for epidemic modeling

### 2.1. Data retrieval, sampling, and pre-processing

**Figure 1:**
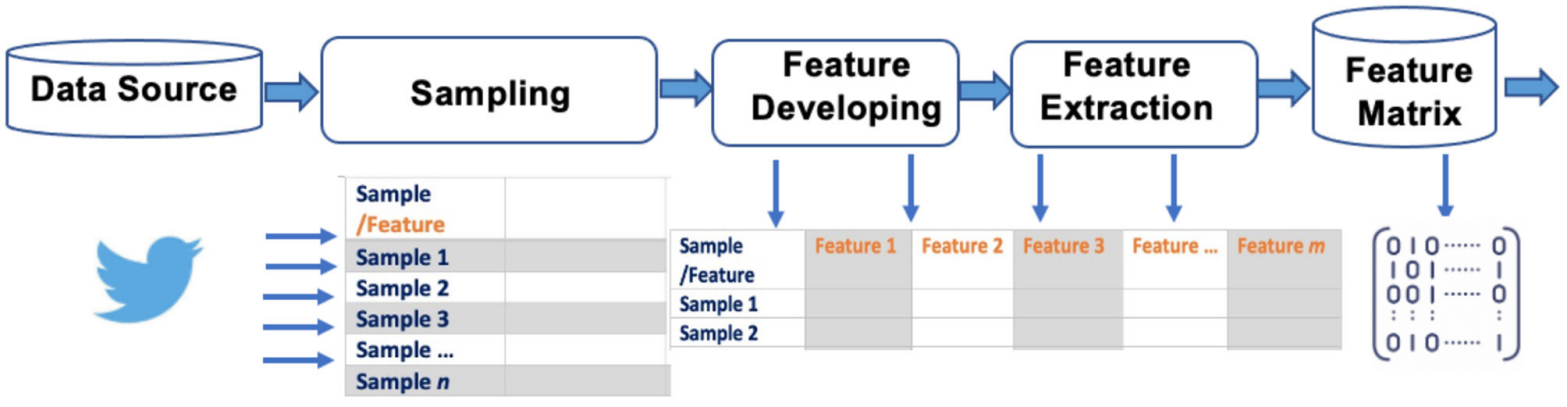
Social media data retrieval, sampling, and pre-processing. using Twitter as an example for online social platforms.

Online discourse data are retrieved and sampled *via* application process interfaces (APIs). Many online platforms, such as Google, Wikipedia, Twitter, Instagram, Facebook, TikTok, have a public API. For instance, COVID-19 Twitter discussion will be acquired *via* the Twitter API. Specific keywords and key phrases related to COVID-19 will be predetermined to the API query, along with other specifications such as frequency and rate of sampling. Because of the sheer volume of COVID-19 discussions, usually a daily random 1% sampling will pull millions tweets per day, adequate for further CSI. Raw data (usually in JSON file format) from API query consist of two components: post body, including mainly the textual data of the post; and post metadata, including posting time, location, ID information (ID, display name, verification status, number of friends, number of followers, etc.), and post virality measures (e.g., numbers of shares, replies, and likes). Raw JSON data are transformed into a dataframe for further mining and analyses. Each row in the dataframe corresponds to a specific tweet post, with both post body and metadata across multiple columns (Figure 1).

### 2.2. Data mining and natural language processing

Once raw data are retrieved and pre-processed, the major task is to transform the unstructured textual data into numeric format for effective analyses. We propose a standardized four dimensions of information to be extracted from each post: (1) time, (2) location, (3) content, and (4) sentiment.

The first two dimensions, time and location will be derived from metadata. However, not all social media users allow sharing their locations, nor would location information always exist in a post. A post can be a general discussion of the health issue. Location data may be determined *via* natural language processing (NLP) of the post. A feasible solution is to develop a rule-based lookup table (LUT) with pre-defined location term database. Depending on the nature and scope of a specific study, the LUT may contain state-level (i.e., names, abbreviations, or other synonyms/indicators of the 51 states, DC, and oversea territories), or county-level (e.g., Mecklenburg County where Charlotte is located) terms. Then, a post is compared with the LUT to determine if there is a match of location terms. A large sample size during the initial API query will ensure adequate spatial coverage. Alternatively, if specific locations are of interest, these locations can be pre-specified in the initial API query (e.g., adding specific location keywords in the query) for sampling.

A third and perhaps the most important dimension is the content (also known as topic or narrative) of the post. A feasible approach is to use LUT with predefined terms to identify specific contents, similar to spatial information identification. For example, “vaccine,” “vaccination,” “inoculation, “shot,” “jab,” “immunization,” “herd immunity” are all be relevant terms of vaccination contents. However, unlike spatial information which can be exhaustively captured by LUT, content information has much more variability and may include terms that are missed in the predetermined LUT. On the other hand, certain terms can have a low specificity. For instance, although “shot” is often interchangeable with “vaccination,” a post mentioning “shot” may not be related to vaccination at all, causing a “false positive” sample of vaccination-related content.

Recent advances in NLP are able to accurately and comprehensively identify content information from textual data (e.g., a social media post, a sentence, a document, and a corpus with multiple documents). Latent Dirichlet allocation (LDA) is a probabilistic-based technique that generates clusters of distributions of words to identify latent topics from input texts. The words in different topics are assumed to have Dirichlet distributions, hence the term LDA. Performance metrics of LDA include perplexity and topic coherence score, which evaluate model predictability and quality of topics, respectively. Outcomes of LDA are the most relevant words in each identified topic. Note that LDA is an unsupervised clustering algorithm, i.e., identified topics come unlabeled from LDA. Therefore, final interpretation and labeling of each topic requires domain knowledge from researchers.

Bidirectional encoder representations from transformers (BERT) is another emerging and powerful NLP technique for topic modeling. The textual data of posts are fed into BERT to generate different levels of embeddings based on the contexts of the word. BERT is constructed from deep neural networks (DNN) with millions of hyperparameters and pre-trained by massive corpus from online text sources including Wikipedia. BERT is able to learn high-level representations of textual data, and cluster reduced embeddings more effectively than probabilistic-based LDA. The clusters are then processed *via* term frequency-inverse document frequency (TF-IDF) to further create topics from clusters. Finally, similar to LDA, domain knowledge is applied to label and interpret identified contents.

In short, different NLP (LUT, LDA, BERT) all fulfill the same objective: further breaking down posts with textual data into more granular, specific contents for further analyses. Certain contents are specifically relevant for epidemic modeling, e.g., discussions on vaccinations and other interventions.

Lastly, sentiment analysis is carried out to evaluate sentiments and/or emotions in the post. Sentiments can be an important indicator of potential health behavioral change, which is crucial for epidemic processes such as infection and vaccination. Depending on the natural of the research, sentiment can be quantified as binary positive or negative, discrete scales (e.g., positive, neutral, or negative) or more granular Paul Ekman six emotion classification and more continuous emotion axes (20–23). Various methods can be used for sentiment analysis, including BERT and ML classification methods (e.g., support vector machine, SVM). In particular, sentiments toward interventions (NPIs and vaccination) can be critical indicators of changes in behavior and epidemic dynamics during the COVID-19 pandemic.

**Figure 2:**
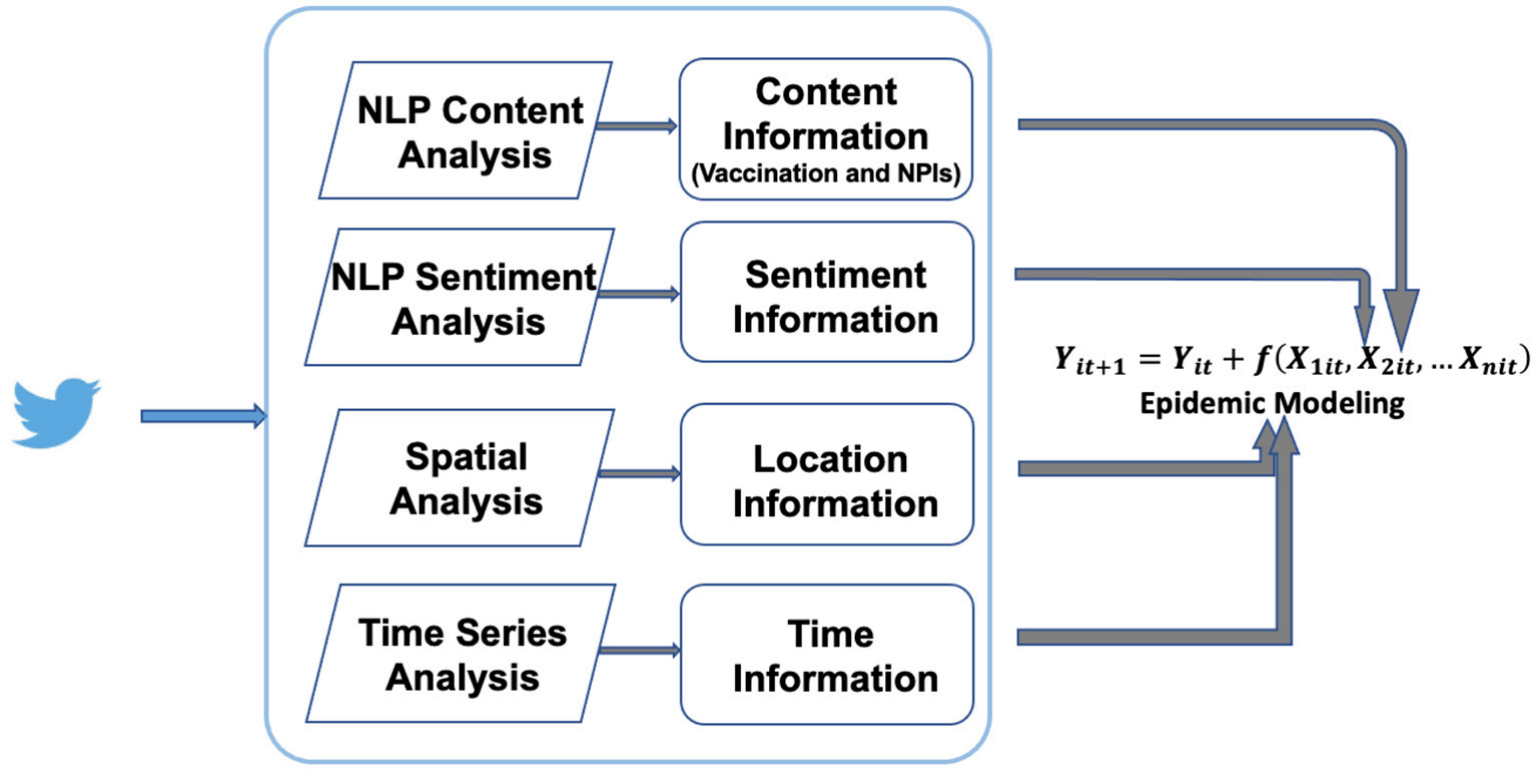
Social media data mining, NLP, and integration with epidemic modeling.

The post-specific dataframe (row as post and columns as the four major dimensions of information) will then be transformed into multiple specific dataframes based on posts' contents, for instance, vaccine-specific, mask mandate-specific, social distancing-specific. The conceptual analytical and NLP framework is presented in Figure 2.

### 2.3. Integrating novel CSI with epidemic modeling: a proposed case study for COVID-19

Once the four dimensions of information – time, location, content, and sentiment – have been retrieved from social media posts, we further recommend the following framework to integrate this novel CSI with epidemic modeling with a case study for COVID-19. This novel CSI significantly increases the amount of information from post contents and sentiments especially on public sentiments toward vaccination and other interventions during the pandemic. We will further extract intervention-specific content, along with sentiments toward these interventions and spatial information. For instance, we will construct a time series of vaccination-related posts (*CV*_*tj*_) at a given location *j*. *CV*_*tj*_ can be either absolute number of posts, or relative percentage in all sampled posts at day *t*. In general, percentage of vaccination-related posts reflects public awareness of the content such as vaccination. The dynamic change of a specific content (e.g., vaccination) percentage reflects the varying degrees of public *awareness* during different phases of the pandemic. In addition, sentiment shifts of the vaccination content topic will also be captured by the sentiment time series, which can be expressed as the percentage of positive or negative sentiment toward vaccination, *SV*_*tj*_. The sentiment time series reflects the dynamic change of vaccination *acceptance* by the public at the location *j*. For instance, vaccination acceptance can be evaluated by positive sentiments or emotions expressed in the posts. Similarly, positive sentiments toward other NPIs (e.g., social distancing, mask-wearing) may indicate increased willingness of compliance with these health policies. These detailed, dynamic characterizations of public awareness and acceptance of vaccination and other NPIs are critical indicators of health decisions and potential behavioral changes (e.g., actively seeking vaccination) during the COVID-19 pandemics. Then, an epidemic model tracks and projects case series *Y*_*t*_ based on current observations and other covariates such as vaccination awareness and acceptance (Figure 2). The functional response of these covariates can be mechanistic (i.e., parameters in SEIR-type model and rules in ABM) or data-driven, discussed below.

The first approach is to use this novel CSI to parameterize and calibrate mechanistic models, including SEIR-type compartment models and more recently introduced agent-based models (ABMs) that tracks detailed behaviors and interactions among individuals. We will compare and evaluate the relationship of content (*CV*_*tj*_) and sentiment (*SV*_*tj*_) time series with traditionally measured health outcomes, such as numbers of reported cases, hospitalizations, and deaths due to COVID-19. By parameterizing vaccination acceptance on these actual health outcomes, it will significantly enhance ABM's ability to further incorporate dynamic behavioral aspects, evaluate effectiveness of vaccination for COVID-19, and predict unintended consequences such as varying vaccination uptake rates across time and location.

Another major category of epidemic modeling is non-mechanistic data-driven models. Our previous study, along with several other studies, have shown that multivariate deep learning models, such as different types of recurrent neural network (RNN) models, can effectively project epidemic dynamics of COVID-19 (18, 19). Depending on different hypotheses, content (*C*) and sentiment (*S*) of interventions can be regarded as input variables that influence observed disease outcomes (*D*), such that *D*_*tj*_ = *f*(*C*_*tj*_, *S*_*tj*_). Alternatively, we could hypothesize no *a priori* influence, i.e., observed disease outcomes and online contents, sentiments toward interventions (e.g., vaccination) can mutually influence each other. Changing health outcomes in different phases of the pandemic can also influence public perceptions of the severity of the COVID-19 pandemic, and consequently alter vaccination acceptance. In this circumstance, multiple time series *D*_*tj*_, *C*_*tj*_, and *S*_*tj*_ are modeled in parallel in RNN to make projections of each time series into the future.

## 3. Discussion

In this paper, we propose a more granular and comprehensive CSI as a critical component in the integrated disease surveillance system through effective data mining on online discourse data during an epidemic such as COVID-19. Social media and other online platforms provide massive data for knowledge discovery through advanced computational techniques, such as NLP. The dynamic changes in public awareness and perceptions toward various interventions, especially COVID-19 vaccination, can be effectively derived from NLP. Exploring these more granular dimensions of information, previously unavailable in traditional infoveillance, should significantly enhance integrative modeling efforts.

This proposed novel CSI framework naturally integrates theoretical foundations of social sciences and technical advances in information and computer science to address an important public health issue: to effectively incorporate cognitive and behavioral aspects into epidemic modeling. Here, we suggest some potential applications of the proposed infoveillance framework. It can effectively identify tipping points in public sentiments toward certain controversial topics, such as vaccination especially in the U.S. Knowing exactly when, where, and how the public will respond to COVID-19 vaccination can be crucial to inform local and national public health agencies to develop health communication strategies to encourage mass immunization and minimize the consequences of preventable cases, hospitalizations, and deaths. In addition, the novel CSI framework can be applied in conjunction with NLP-based misinformation detection methods to monitor surges of vaccination-related misinformation. This CSI framework could also evaluate responses and perceptions of different populations (e.g., race/ethnicity, age, or other social determinants of health) to specific types of interventions.

While social media provide large volumes of public discourse data on diseases to characterize public responses, sampling bias may still occur due to the observational study nature of passive infoveillance. Users of online platforms such as social media may not be adequately representative of the target population. Therefore, active participatory studies, such as randomized surveys, can complement this novel CSI *via* social media analytics.

## Data availability statement

The original contributions presented in the study are included in the article/supplementary material, further inquiries can be directed to the corresponding author.

## Author contributions

All authors listed have made a substantial, direct, and intellectual contribution to the work and approved it for publication.
